# Sleep in the Human Hippocampus: A Stereo-EEG Study

**DOI:** 10.1371/journal.pone.0000867

**Published:** 2007-09-12

**Authors:** Fabio Moroni, Lino Nobili, Giuseppe Curcio, Fabrizio De Carli, Fabiana Fratello, Cristina Marzano, Luigi De Gennaro, Franco Ferrillo, Massimo Cossu, Stefano Francione, Giorgio Lo Russo, Mario Bertini, Michele Ferrara

**Affiliations:** 1 Department of Psychology, University of Rome La Sapienza, Roma, Italy; 2 Centre of Epilepsy Surgery “C. Munari”, Center of Sleep Medicine, Niguarda Hospital, Milano, Italy; 3 Institute of Bioimaging and Molecular Physiology, Section of Genova, National Research Council, Genova, Italy; 4 Sleep Disorders Center, University of Genoa, Genoa, Italy; 5 Department of Internal Medicine and Public Health, University of L'Aquila, L'Aquila, Italy; Lund University, Sweden

## Abstract

**Background:**

There is compelling evidence indicating that sleep plays a crucial role in the consolidation of new declarative, hippocampus-dependent memories. Given the increasing interest in the spatiotemporal relationships between cortical and hippocampal activity during sleep, this study aimed to shed more light on the basic features of human sleep in the hippocampus.

**Methodology/Principal Findings:**

We recorded intracerebral stereo-EEG directly from the hippocampus and neocortical sites in five epileptic patients undergoing presurgical evaluations. The time course of classical EEG frequency bands during the first three NREM-REM sleep cycles of the night was evaluated. We found that delta power shows, also in the hippocampus, the progressive decrease across sleep cycles, indicating that a form of homeostatic regulation of delta activity is present also in this subcortical structure. Hippocampal sleep was also characterized by: i) a lower relative power in the slow oscillation range during NREM sleep compared to the scalp EEG; ii) a flattening of the time course of the very low frequencies (up to 1 Hz) across sleep cycles, with relatively high levels of power even during REM sleep; iii) a decrease of power in the beta band during REM sleep, at odds with the typical increase of power in the cortical recordings.

**Conclusions/Significance:**

Our data imply that cortical slow oscillation is attenuated in the hippocampal structures during NREM sleep. The most peculiar feature of hippocampal sleep is the increased synchronization of the EEG rhythms during REM periods. This state of resonance may have a supportive role for the processing/consolidation of memory.

## Introduction

The hippocampal formation is crucial for the storage of facts, episodes and spatial informations, the so-called declarative memories [1], [2]. In recent years a series of papers have provided electrophysiological evidence pointing to the involvement of the hippocampus in memory processing also during sleep. For example, high order replay of waking activity has been observed in the hippocampus during both SWS and REM sleep [3]–[5]. In this replay, a temporally sequential firing order across multiple cells is recaptured during sleep, possibly reflecting the consolidation of episodic memory traces [6]. Hippocampal reactivations during SWS, correlated with an improvement in spatial memory performance, have been reported also in humans [7]. However, despite the surge of interest in the coordinated hippocampal-cortical activity during sleep, not much is known about the exact features of the human sleep recorded from the hippocampus.

Non-invasive methods such as the electroencephalogram (EEG) still provide most of the current data about the human sleep. Based on these data, the two-process model of sleep regulation posits that sleep propensity exponentially rises during waking and declines during sleep [8], [9]. Slow-wave activity (SWA) in non-REM (NREM) sleep, i.e. the EEG power in the 0.75–4.5 Hz range quantified by spectral analysis, is considered a marker of NREM sleep intensity and the electrophysiological correlate of a sleep-wake dependent process (Process S) underlying sleep homeostasis [8], [9]. Do this characteristic and basic feature of human scalp-recorded sleep EEG also apply to sleep recorded from deep brain structures, such as the hippocampus?

Indubitably, the EEG spatial resolution is insufficient to show physiological processes in deep brain structures. Clinical approaches, however, sometimes allow invasive recordings to be taken from the human brain, mainly in epileptic patients. These recordings can provide unique insights into brain functions, including sleep. Although there have been previous reports on human sleep recorded from subcortical regions, they were mainly focused on the analysis of single EEG bands during specific sleep stages [10]–[14]. Moreover, some of these studies did not record sleep directly from deep structures, but used electrocorticography with subdural electrodes [15], [16] or with foramen ovale electrodes [17]–[18].

The aim of the present study is thus to provide a detailed and systematic analysis of human sleep directly recorded from the hippocampus and the neocortex in a group of epileptic patients undergoing presurgical examinations. To this aim, we evaluated the time course of classical EEG frequency bands during the first three NREM-REM sleep cycles of the night, considering both scalp EEG and intracerebral stereo-EEG (SEEG) recordings of the same subjects.

## Materials and Methods

### Subjects

Five patients (3 females, mean age: 35.8±6.1 years, age range: 27–43 years, see Table 1) with pharmacoresistant focal epilepsy underwent an individualized investigation with stereotactically implanted intracerebral multilead electrodes for a careful definition of the epileptogenic zone for surgical purposes (see [19] for details on SEEG methodology). In Table 1 a summary of demographic and clinical information for each patient is reported. None showed features indicative of hippocampal sclerosis and SEEG revealed that seizures originated outside the temporal mesial structures.

**Table 1:** Demographic, MRI findings and clinical information for each patient.

Patient	Gender	Age (years)	Medications (mg/day)	MRI findings	SEEG				
					Side	Sample lobes	Neocortex*	Hippocampus*	Epileptogenic Zone**
**1**	**M**	40	Carbamazepine (800)	Unrevealing	Left	FT	Superior F gyrus	Left Anterior-Central	Middle F gyrus
			Topiramate (300)						
**2**	**F**	27	Carbamazepine (1600) Vigabatrin (3000) Levetiracetam (3000)	Right O cortical dysplasia	Right	TPO	Supramarginal gyrus	Right Central-Posterior	TO basal junction
**3**	**M**	43	Oxcarbazepine (1500) Topiramate (600) Clonazepam (1.5)	Right C-T-P polymicrogyria	Right	FCPT	Inferior F gyrus	Right Posterior	First T gyrus
**4**	**F**	34	Carbamazepine (1200) Levetiracetam (3000) Phenobarbital (100)	Left P-O ischemic lesion	Left	TOP	Secod T gyrus	Left Posterior	Fusiform gyrus
**5**	**F**	35	Oxcarbazepine (1200) Barbexaclone (100) Topiramate (400)	Left T-O periventricular heterotopia	Left	TOPC	Supramarginal Gyrus	Left Central-Posterior	P cingulated gyrus

C = central; F = frontal; O = occipital; P = parietal; T = temporal.

* Indicates the position of the SEEG derivations submitted to sleep EEG analysis.

** Indicate the site of origin of the seizures.

Sleep was recorded five days after electrode implantation. Sleep recordings of the first adaptation night were not considered in the analyses. Before intracerebral electrodes implantation, patients gave their written informed consent for participation in this research study and for publication of data. The protocol was approved by the Ethical Committee of the Niguarda Cà Granda Hospital.

### Electrodes placement and EEG/SEEG recordings

**Figure 1:**
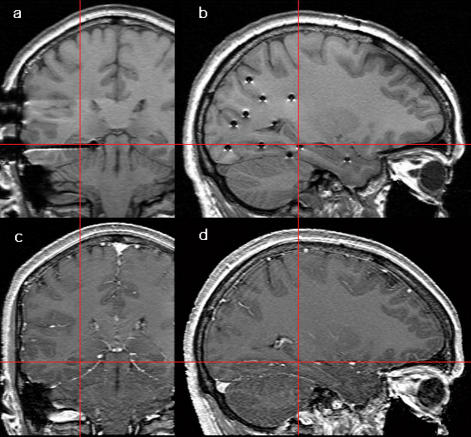
Magnetic resonance imaging (MRI) scan of intracranial electrodes implanted into the medial temporal lobe (a and b panels, coronal and sagittal views, respectively). Red line cross indicates the location of the two electrode contacts within the hippocampus. C and d panels show the MRI scan of the same locations before the electrode implant.

Stereo-EEG activity was recorded from platinum-iridium intracerebral electrodes, with a diameter of 0.8 mm, a contact length of 2 mm and a intercontact distance of 1.5 mm. Each subject had at least two electrode contacts which could be localized unequivocally within the hippocampus and at least two contacts within the neocortex. The individual placement of electrode contacts was ascertained by post-implantation magnetic resonance imaging (MRI) scans (for individual location details see Table 1). Figure 1 (a, b) shows an example of MRI coronal and sagittal scans of intracranial electrodes implanted into the hippocampus.

Scalp EEG activity was recorded from two platinum needle electrodes placed during surgery at “10–20” positions Fz and Cz on the scalp. Electroocular activity was registered at the outer canthi of both eyes and submental electromyographic activity was acquired with electrodes attached to the chin.

EEG and SEEG signals were recorded using a 24 channels ambulatory system recording (XLTEK, Trex™) with a sampling rate of 512 Hz.

### Procedure

The study began at 8 p.m. Patients were connected to the polygraph and the recording started. Then patients were free to go to their room and decide by themselves when going to sleep. The following morning at 7.30 a.m. patients were disconnected and data were downloaded from the portable device memory card and stored on the hard disk of a computer. During the study patients continued taking their standard doses of anticonvulsant medications (for details see Table 1).

### Data Analysis

Acquisition files were converted to EDF (*European Data Format*) to be handled with a MatLab software (MatLab 7.0, The Matworks, Inc.). This software allowed us to modify montage settings and set filters to the signal. We used a bipolar montage between contiguous electrode contacts and between Fz-Cz scalp electrodes, EOG and EMG derivations. EEG and SEEG channels were 0.1–30 Hz band pass filtered, EOG channel was 0.16–15 Hz band pass filtered and EMG channel was 5–150 Hz band pass filtered. The same software allowed us to score the sleep stages according to the standard criteria [20], to remove artefacts and to run power spectral analysis.

Sleep was scored in 20 s epochs starting from 20 min before the first stage 2 epoch. During sleep scoring, EEG periods with interictal spikes and pathological EEG signals were triggered in order to remove them from the subsequent analysis. Power spectra were computed by a *Fast Fourier Transform* routine for 4 s periodograms averaged in 20 s epochs, resulting in a frequency resolution of 0.25 Hz. Frequency range was 0.5–30 Hz. The bands boundaries were as follows: very low frequencies (VLF, encompassing the slow oscillation range) 0.5–1 Hz; delta 1.1–5 Hz; theta 5.1–8 Hz; alpha 8.1–12 Hz; sigma 12.1–15 Hz; beta 15.1–30 Hz.

## Results

### Time course of EEG bands power spectra across the first three sleep cycles

**Figure 2:**
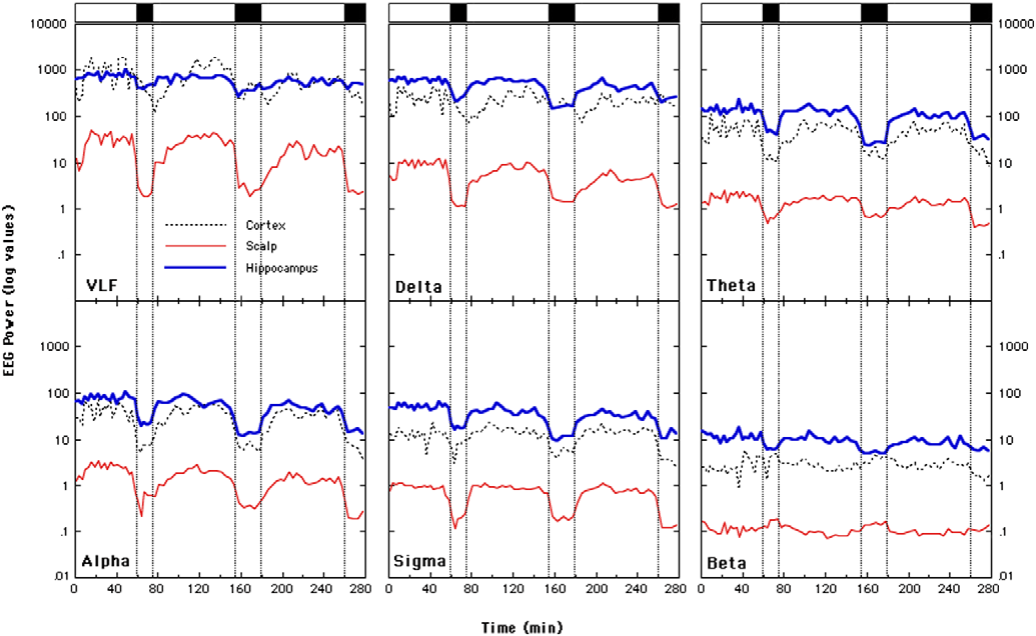
Time course of EEG power in the 0.5–30.0 Hz frequency range across the first three NREN-REM sleep cycles. REM periods are indicated by the black rectangles on top of the figure. Data are expressed in a logarithmic scale. Scalp recordings have been obtained by means of a bipolar (Cz-Fz) montage. Intracranial (cortex and hippocampus) stereo-EEG recordings have been obtained by a bipolar montage between two contiguous contacts of a platinum-iridium microwire. Very low frequency range (VLF, 0.5–1.0 Hz); delta frequency range (1.1–5.0 Hz); theta frequency range (5.1–8.0 Hz); alpha frequency range (8.1–12.0 Hz); sigma frequency range (12.1–15.0 Hz); beta frequency range (15.1–30.0 Hz).

As a first step we sought to determine the time course of EEG power for each band across the night. To this aim, we chose the first three NREM-REM sleep cycles, since they were the minimal common number of sleep cycles. Log values of EEG-SEEG power for each band in the first three sleep cycles are reported in Figure 2.

The EEG frequencies up to 15 Hz (delta, theta, alpha and sigma bands) all showed a clear drop of power coincident with the onset of each REM period. In general, this was true for the scalp recordings, as well as for the neocortical and hippocampal SEEG recordings. Regarding the VLF range, however, such a decrease of power was largely less discernible in the hippocampus (Figure 1, thick blue line), that showed a smoother time course of SEEG power across the whole night irrespective of the succession of NREM-REM periods. On the other hand, the hippocampal SEEG power drop in the delta band (1.1–5 Hz) during REM periods was clearer, although it was less steep than in the scalp recordings. It should be emphasized that the progressive decrease of delta power across sleep cycles, predicted by the two-process model of sleep regulation, was unambiguously present in all the recordings, although it was slightly more clear-cut in the scalp than in the deep brain derivations.

Theta, alpha and sigma activity showed a similar behavior, with maximal power in the hippocampal recordings and minimal power over the scalp, and a generalized drop of power in concomitance with REM periods. On the other hand, beta power showed distinct behaviors in the hippocampal recordings compared to the scalp and the neocortex. In fact, while in the latter two derivations beta power seems to increase during REM periods, in the former beta power seems to be higher during NREM sleep, decreasing during REM sleep.

Three phenomena seem to emerge from this general description of hippocampal sleep features: i) a flattening of the VLF time course across sleep cycles, with relatively high levels of power even during REM sleep; ii) a very high SEEG delta power, exceeding that in the neocortical recordings; iii) a decrease of power in the beta band during REM sleep, at odds with the typical increase of power seen both in the neocortex and in the scalp recordings.

**Figure 3:**
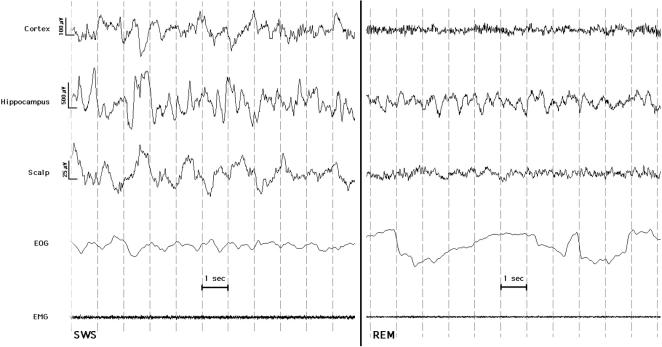
Examples of EEG patterns recorded from intracranial (cortex and hippocampus) and scalp electrodes during slow wave sleep (SWS) and rapid eye movement sleep (REM). Electrooculogram (EOG) and chin electromiogram (EMG) are also reported. See text for other methodological details about recordings. A clear slow rhythm is present in the hippocampal recording also during REM sleep.

These phenomena seem to point to an increased general synchronization of the SEEG rhythms in the hippocampus, especially during REM periods. In fact, during REM sleep the intrahippocampal recordings clearly show a low-delta frequency rhythm, reported by others around 1.5 Hz using foramen ovale recordings [17], and easily discernible by visual inspection (see Figure 3), accompanied by a peculiar decrease of beta power.

**Figure 4:**
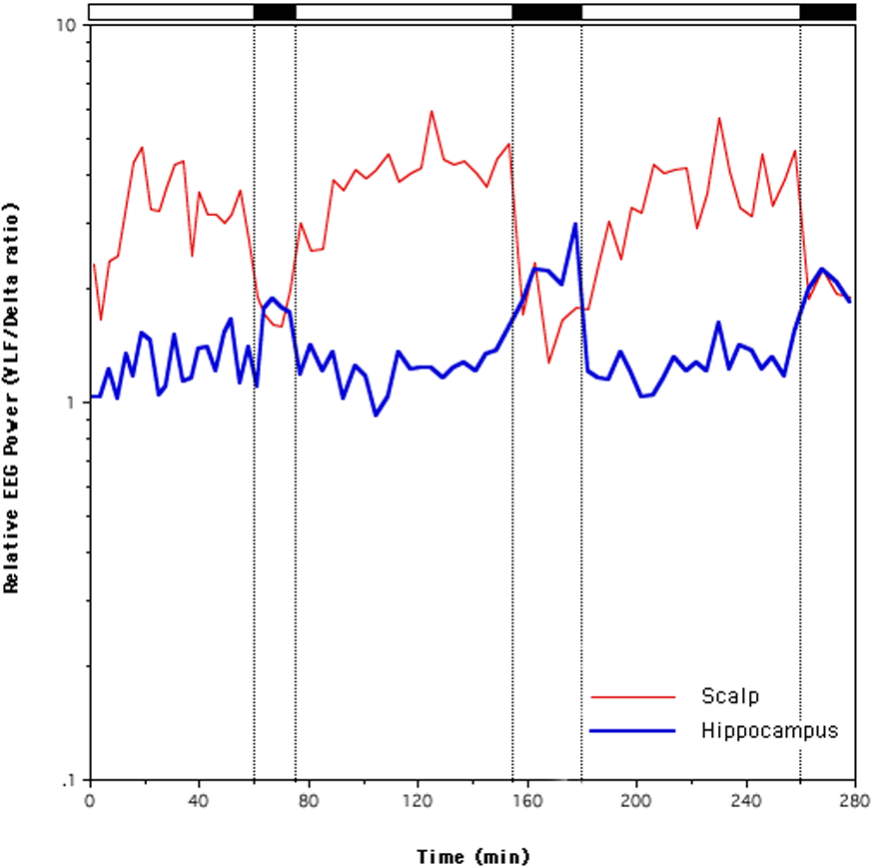
Time course of the very low frequency/delta power ratio (0.5–1.0/1.1–5.0 Hz) across the first three NREM-REM cycles, separately for scalp and hippocampal recordings. REM periods are indicated by the black bars on top of the figure.

**Figure 5:**
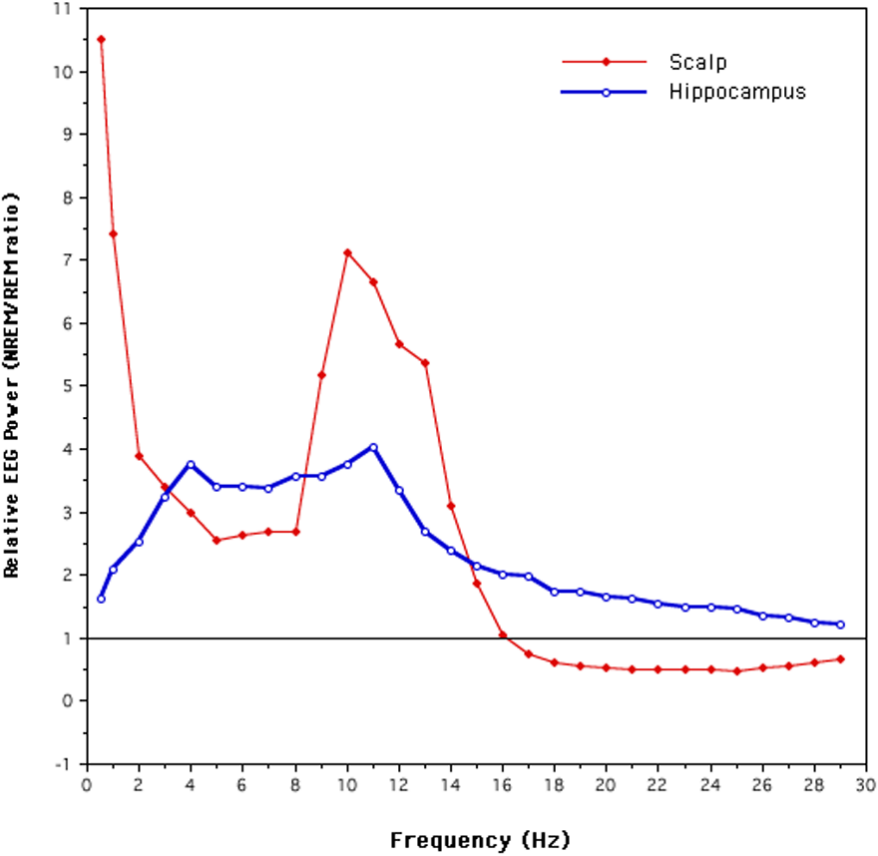
NREM/REM EEG power ratio in the entire (Hz by Hz) 0.5–30 Hz frequency range of the whole night, separately for scalp and hippocampal recordings. The horizontal line indicate the mean REM sleep EEG power (value = 1). Mean values close to this line indicate that NREM and REM sleep power are equivalent in that frequency bin.

An opposite behavior of the two low frequency ranges during NREM and REM sleep becomes even clearer when we plot the time course of their power ratio (0.5–1.0/1.1–5.0 Hz) across the first three NREM-REM cycles, separately for scalp and hippocampal recordings (Figure 4). Figure 4 clearly shows that VLF/delta power ratio has a reciprocal relation in NREM and REM sleep. In fact, values are very high in the NREM scalp recordings and very low during REM periods, confirming a clear predominance of EEG power in the very low frequencies (up to 1Hz) during NREM sleep. On the other hand the opposite holds true for the hippocampus where, even in the frame of lower absolute power values compared to the scalp, a sharp increase of relative power is visible during REM sleep compared to NREM. This result is due to the persistence of relatively high levels of SEEG power in the very low frequencies even during REM sleep in the hippocampus, as already shown in Figure 2. This becomes even clearer when we plot the relative NREM/REM EEG power in the entire 0.5–30 Hz frequency range (see Figure 5). Figure 5 shows that hippocampal SEEG power during NREM and REM sleep are very close at the edges of the considered frequency range (where relative values are close to 1). However, this is due to opposite behaviors of the slow and fast rhythms: i) SEEG power in the very low frequencies remains high during REM sleep in the hippocampus; ii) the unexpected decrease of beta power during REM sleep gives rise to a relative preponderance of beta during NREM sleep, at odds with the typical decrease of NREM beta power seen in the scalp recordings. On the other hand, the relative power in the scalp recordings shows, as usual, the highest NREM EEG power in the delta band, a second peak in the alpha-sigma range, and a drop of power in the beta range.

**Figure 6:**
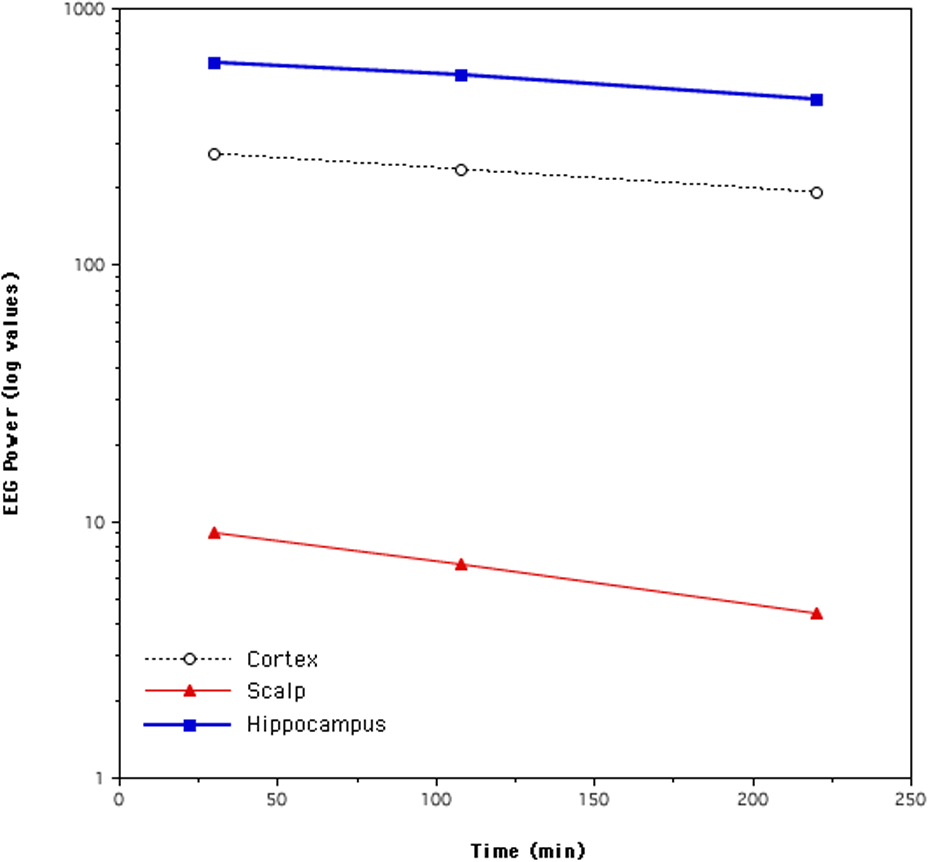
Linear decrease of delta power (1.1–5.0 Hz) across the first three sleep cycles in the cortical, hippocampal and scalp recordings. Values are expressed in a logarithmic scale.

Lastly, we evaluated the kinetics of delta power (1.1–5 Hz) across the three considered sleep cycles, separately for cortical, hippocampal and scalp recordings. As showed in Figure 6, delta power linearly decreased across the night in all the recordings (scalp: y = 9.6718–2.4428e-2x, R^2^ = 0.996; cortex: y = 284.29–0.42020x, R^2^ = 0.998; hippocampus: y = 642.17–0.90639x, R^2^ = 0.993). An exponential fit was not feasible, given the small number of data point available.

## Discussion

Here we showed that hippocampal sleep exhibits some very peculiar, distinctive features during both NREM and REM sleep periods, as revealed by power spectral analysis. More specifically:

- NREM sleep in the hippocampus is characterized by a lower relative SEEG power in the slow oscillation range (up to 1 Hz) compared to the scalp EEG. In other words, while in the scalp recordings EEG power in the very low frequencies outweigh delta power, the hippocampal recordings show a kind of power balance between the slow oscillation and the delta frequency ranges due to a decreased power in the very low frequencies.
- SEEG power in the lower frequencies remains quite stable also during REM sleep, failing to show the typical drop of power as in the cortical and scalp recordings.
- During REM sleep we found a decrease of beta power in the hippocampal recordings.

Together, these findings seem to indicate that during REM sleep the hippocampus show a generalized tendency to EEG synchronization.

Nevertheless, our analysis also revealed that sleep in the hippocampus shares some basic characteristics with the long known scalp recorded sleep. In fact, the well recognized drop of delta power in coincidence with the onset of REM sleep, as well as the progressive decrease of delta power across cycles are distinctive features also of the hippocampal sleep.

### Time course of delta SEGG power across sleep cycles

The kinetics of NREM delta EEG power is of particular interest in sleep physiology mainly because these frequencies are homeostatically regulated in response to prior waking duration [8], [9]. The level of EEG slow-wave activity (SWA) is determined by the duration of prior sleep and waking. SWA is a marker of NREM sleep intensity and may serve as an indicator of sleep homeostasis. In fact, SWA is high in the early part of the sleep period, when sleep need is greatest, and exhibits an exponential decline across the subsequent NREM sleep episodes. Interestingly, in the present study the decline of SWA across the sleep episode, essentially reflecting recovery processes during sleep, was similar in the scalp and in the depth SEEG cortical and hippocampal recordings. This result seems to indicate that the homeostatical properties of delta EEG power are preserved also in the subcortical structures, as already shown in cats [21]. As a matter of fact, it has been reported that, following a 12-hour period of sleep deprivation, hippocampal slow wave activity was enhanced at the beginning of the recovery period and gradually declined thereafter [21]. The next step will be to assess whether or not delta activity increases in the hippocampus as a function of prior sleep deprivation, as predicted by the two-process model whether hippocampal slow wave activity is homeostatically regulated also in humans.

The neural mechanisms underlying the increase in SWA with increasing sleep pressure are still unknown. However, there are several recent evidence showing that NREM delta activity can be regulated locally in the cerebral cortex [22]–[24]. Moreover, a PET-EEG study demonstrated that the increase in SWA is correlated with a regional cerebral blood flow decrease in neocortical areas [25]. These studies suggest that SWA is directly affected by plastic changes in local (cortical) circuits. If such relationship between use-dependent local neural changes and delta activity in NREM sleep also applies to the hippocampus, an increase in SWA after declarative (hippocampus dependent) learning should be expected. This hypothesis can be testable in the near future. In any case, it should be kept in mind that such an increment in hippocampal SWA may indicate the presence of local use-dependent processes, but it does not imply that other experience-dependent processes cannot take place outside of use-dependent activity during sleep.

### Behavior of the hippocampal very low frequencies during NREM sleep

Slow oscillations are generated in the neocortex and reflect widespread up and down states of network activity. The down state is a phase of hyperpolarization with neuronal silence, followed by a depolarization phase or up state characterized by intense synaptic activity and neuronal firing [26]. Due to its synchronizing influence on neuronal activity within the neocortex and in the dialogue with thalamic and hippocampal structures, it has been hypothesized that the slow oscillation may underly the memory consolidation processes during sleep [27], [28]. In the present work, we observed that EEG power in the frequency range of scalp recorded slow oscillation (up to 1 Hz) shows its maximal values in NREM sleep, being largely higher than EEG power in the delta range (1.1–5 Hz). However, in the hippocampal recordings we found a relatively lower SEEG power in the SO range during NREM sleep, whose power only slightly exceeded that in the delta band. This differential behavior of very low frequencies in the cortical and hippocampal recordings was not predicted. A relative decrease of power in this range can be accounted for by a decreased number of SO cycles (*down-up* states), as well as by a decreased amplitude of those cycles. However, at present we can not elucidate this point since in this work we did not count the cycles or the amplitude of the slow oscillations.

Although it is known that slow oscillation originates at a definite cortical site and spreads over the scalp surface within a few hundred milliseconds [29], whether or not such propagation also exists from the neocortex to subcortical sites in humans is still unclear. In other words, the SO activity recorded in the hippocampus could be due to the propagation of the neocortical SO to the hippocampus or, alternatively, to an intrinsic very slow rhythm generated within the hippocampus or in the parahippocampal regions. In both cases, however, the orderly propagation of correlated activity along connected pathways during sleep may lead to synaptic consolidation [28], [30] or downscaling [31]. It could be speculated that the relatively lower SEEG power in the VLF range during hippocampal NREM sleep may be linked to a relatively high level of activation in the hippocampus. Although this may appear contradictory with the hypothesis that the slow oscillation underlies memory consolidation processes, it should be noticed that in the study reporting a reactivation of hippocampal activity in humans [7], a learning-dependent increase in regional cerebral blood flow was observed during NREM sleep in the hippocampus, which actually means less synchronization, and consequently lower slow oscillation activity. In agreement with these findings, neurophysiological data in animals [32] and in humans [33] indicate the persistence of an important activity in the hippocampus during SWS. Finally, our result of an independent behavior of the VLF in the hippocampus compared to the neocortex is partly at odds with the parallel variations of SWA in the same structures reported in a pioneering single case report [34]. There are several potential causes for these differences: type of recordings (SEEG vs foramen ovale electrodes); electrode locations (hippocampus vs parahippocampal gyrus); EEG band boundaries; epileptogenic zones (as reported in Table 1 vs right mediobasal temporal lobe); number of subjects (5 vs 1).

Recent studies in animal models have provided a complex picture of the neocortical-hippocampal SO-triggered dialogue. In fact, it has been reported that some hippocampal cells (namely granule cells and CA3 and CA1 pyramidal cells) lacked bimodality, yet they were influenced by the cortical slow oscillation in a region-specific manner [35]. On the other hand, CA1 interneurons did show up and down states (UDS) and their membrane potentials were phase-locked to neocortical UDS with a small delay [36]. Most recently, dentate gyrus granule cells showed clear UDS modulation that was phase locked to cortical UDS with a short delay. In contrast, CA3 pyramidal neurons showed mixed UDS modulation, such that some cells were depolarized during the cortical up state and others were hyperpolarized [37]. These results provide strong evidence of cortico-hippocampal interaction and suggest that neocortical activity drives hippocampal interneurons during UDS. At the same time, though, a differential and complicated functional connectivity between neocortex and hippocampal subfields during slow oscillations seems to emerge.

Our finding of a relative decrease of hippocampal SEEG power in the very low frequency range during NREM sleep, may be interpreted as support to the idea that cortical SO arrives attenuated in the hippocampal structures. However, further studies on the phase-locking of cortical and hippocampal slow oscillations are needed to elucidate this issue.

### General synchronization of SEEG rhythms in the hippocampus during REM sleep

The most striking and peculiar feature of hippocampal sleep seem to be the general synchronization of the SEEG rhythms during REM sleep, as indicated here by the relative increase of power in the slow frequency range accompanied by a decrease of power in the beta range. This result is at odds with the reported reductions of scalp recorded power values in the delta-theta frequency range along with the prevalence of beta power typically seen at REM onset [38].

Hippocampal rhythmic slow activity (RSA or theta) is a distinctive feature of REM sleep of rodents, carnivores, primates and humans [11], [39]–[41]. A slower (1–3 Hz) rhythmic hippocampal oscillation has also been reported in humans, suggesting that different types of hippocampal rhythmic oscillations may coexist in human hippocampus during REM sleep [17]. Such a dominant sustained frequency in the human hippocampus (around 2–2.75 Hz in the present study) may also be viewed as a slower theta activity, which is consistent with a larger brain size. It has been proposed that hippocampal RSA provides a mechanism for the consolidation of new memories and for weakening/erasing older ones during REM sleep [42], [43]. Our data lend some indirect support to this hypothesis by further showing that human hippocampal sleep is characterized by an increased SEEG synchronization. It will be interesting to assess whether or not declarative learning exerts some effect on such synchronization of hippocampal SEEG activity. In fact, if this activity is linked to the memory consolidation processes, it would result further increased after declarative learning compared to control conditions (no learning, procedural learning).

Recently an fMRI study reported during phasic REM sleep the activation of a brain circuit comprising limbic, hippocampal and parahippocampal structures [44]. Synchronized hippocampal-parahippocampal (but also thalamic and limbic) activity in REM sleep may well be associated with some kind of memory processing, but also create intense emotional dream contents, as suggested by others [45], [46].

Our finding of a relative increase of power in the very low frequency range during REM sleep point to the possibility that such rhythm is intrinsically generated within the hippocampus, being completely independent from the cortical SO of NREM sleep. This very state of resonance during REM sleep may have a supportive role for the consolidation of (emotional) memory.

### Concluding remarks

Hippocampal sleep features have been studied here in epileptic subjects undergoing presurgical evaluations. Since epilepsy has been proposed as a disorder of synchronization, some of the effects reported may be viewed as a manifestation of such pathology. Although the generalizability of the results obtained in peculiar subjects to the general population may be always questionable, we believe that our findings reflect normal brain activity. In fact the subjects included in this study did not show any lesion or epileptic focus within the hippocampus or parahippocampal regions. Moreover, the analyzed sleep epochs were free from interictal epileptic discharges in all the recorded channels. In any case, the presence of epileptic spikes during REM sleep is quite rare.

However, several issues remains to be elucidated, such as the reason for the decreased hippocampal power in the slow oscillation range during NREM sleep. A direct evaluation of the hippocampal origin of the slow rhythms reported during REM sleep is also in order. These points may be possibly enlighted by the specific analysys of the phase relations between neocortical and hippocampal slow oscillations in NREM and REM sleep, considering the single slow oscillations as traveling waves (as in [29]). Finally, the links between EEG rhythms and memory processes would benefit from the careful investigation of the relationships between slow (SO) and fast phasic activities (sleep spindles, sharp waves) during sleep.
